# Neurological risk in urgent valve replacement cardiac surgery for infective endocarditis with cerebrovascular complications - a case series

**DOI:** 10.1186/1749-8090-10-S1-A56

**Published:** 2015-12-16

**Authors:** Apoorva Ballal, Sara Mirza, Jaishanker Raman

**Affiliations:** 1 University of Aberdeen Medical School, Foresterhill, Aberdeen AB24 2ZD, UK; 2 Department of Cardiovascular Thoracic Surgery, Rush University Medical Centre, 1645 W Jackson Boulevard 310, Chicago, IL 60612, USA

## Background/Introduction

The indications for urgent valve replacement in patients with infective endocarditis (IE) include uncontrolled infection, recurrent emboli, valve dysfunction and cardiac failure. The optimal timing of this surgery when IE is complicated by cerebrovascular events (such as cerebral infarction or haemorrhage) is controversial, largely due to the escalated risk of perioperative intracranial bleeding, given that the patient is fully anticoagulated and on cardiopulmonary bypass for the procedure. While current guidelines suggest waiting at least 2 weeks between the diagnosis of stroke and surgery, with a deteriorating clinical condition valve surgery might be beneficial if done earlier.

## Aims/Objectives

The aim of this study was to investigate clinical and neurological outcomes with early valve replacement surgery (within 10 days) following a stroke, in a series of consecutive patients with IE.

## Method

This was a single-centre retrospective analysis of 17 out of 47 patients requiring surgery for IE between 2011 and 2014 at a tertiary cardiac centre, who also had either ischemic or haemorrhagic cerebrovascular complications. Time on bypass and hence on full anticoagulation was taken as one measure, amongst others, of surgical risk. Neurological risk and outcomes pre- and post-operatively were assessed using the National Institute of Health Stroke Scale and the Glasgow Outcome Score (GOS).

## Results

All 17 patients underwent cardiac valve surgery within 10 days of the diagnosis of stroke (14 patients at,/= 7 days). Mortality within the immediate postoperative period was 12%. The remaining 15 patients exhibited good neurological recovery in the immediate post-operative period with a GOS score of 3 or above. Further analysis of followup where available demonstrated significant improvements in neurological function with several patients returning to full functional capacity and independent living. There was no correlation between duration of cardiopulmonary bypass and neurological outcomes.

## Discussion/Conclusion

While the sample size of 17 patients in this study is small, largely due to the relative rarity of IE requiring cardiac surgery and also having a cerebrovascular complication, it does show that urgent valve replacement surgery in this clinical situation is possible within the acute period following the intracranial event without compromising neurological function and can be potentially life saving

